# Genetics of scleroderma: implications for personalized medicine?

**DOI:** 10.1186/1741-7015-11-9

**Published:** 2013-01-11

**Authors:** Shervin Assassi, Timothy RDJ Radstake, Maureen D Mayes, Javier Martin

**Affiliations:** 1 Division of Rheumatology and Clinical Immunogenetics, The University of Texas Health Science Center at Houston, 6431 Fannin Street, Houston, Houston, Texas, TX 77030, USA; 2 Department of Rheumatology & Clinical Immunology, University Medical Center Utrecht, Heidelberglaan 100, Utrecht, 3584 CX The Netherlands; 3 Instituto de Parasitología y Biomedicina López-Neyra, IPBLN-CSIC, Parque Tecnológico Ciencias de la Salud, Avenida del Conocimiento, Armilla, Granada, 18100 Spain

**Keywords:** systemic sclerosis, scleroderma, genetic, biomarker, severity

## Abstract

Significant advances have been made in understanding the genetic basis of systemic sclerosis (scleroderma) in recent years. Can these discoveries lead to individualized monitoring and treatment? Besides robustly replicated genetic susceptibility loci, several genes have been recently linked to various systemic sclerosis disease manifestations. Furthermore, inclusion of genetic studies in design and analysis of drug trials could lead to development of genetic biomarkers that predict treatment response. Future genetic studies in well-characterized systemic sclerosis cohorts paired with advanced analytic approaches can lead to development of genetic biomarkers for targeted diagnostic and therapeutic interventions in systemic sclerosis.

## Background

Systemic sclerosis (SSc or scleroderma) is a multisystem, uncommon disease characterized by fibrosis in skin and internal organs, immune dysregulation, and vasculopathy. Its pathogenesis remains poorly understood but there is a growing body of evidence implicating in part genetic factors. However, the genetic basis for SSc is defined by multiple genes that have only modest effect on disease susceptibility [1,2]. Moreover, the disease is thought to arise from an interaction between genetic factors and environmental triggers.

SSc is subdivided into limited and diffuse types based on the extent of skin involvement [3]. Furthermore, SSc can be sub-grouped based on the presence of non-overlapping autoantibodies that are associated with various disease manifestations [4]. The standardized mortality ratio of patients with SSc is 3.5 [5] which is higher than most other rheumatic diseases. Reliable predictors of disease course and therapeutic options are very limited. Genetic data are not time-dependent and do not change over the course of disease; thus they are attractive candidates for development of predictive biomarkers. In this review, we will examine the implication of recent discoveries in SSc genetics for drug development and identification of predictive biomarkers.

### Recent advances in SSc genetics

Case-control candidate gene studies have identified several robust SSc susceptibility loci that have been confirmed in subsequent independent studies (reviewed in [1,2]). The majority of these genes such as *IRF5 *[6], *STAT4 *[7], *BANK1 *[8] and *BLK *[9] belong to pathways involved in immune regulation. Furthermore, three genome wide association studies (GWAS) allowed unbiased genetic profiling of patients with SSc [10-12]. These studies have confirmed genes in the major histocompatibility complex (MHC) as the strongest susceptibility loci. Furthermore, a GWAS follow-up study confirmed that *HLA-DQB1, HLA-DPA1/B1*, and *NOTCH4 *associations with SSc are likely confined to SSc specific auto-antibodies [13].

Multiple non-MHC susceptibility loci also have been identified in the above-mentioned studies. As shown in Table 1, the most robust associations are in genes related to innate immunity, as well as B- and T-cell activation. For example, *IRF5 *belongs to a family of transcription factors in the type I interferon pathway which is an important component of the innate immunity, whereas *CD247 *encodes the T-cell receptor zeta subunit modulating T-cell activation. The majority of these gene variants are also risk loci for other autoimmune diseases, especially for systemic lupus erythematosus (SLE) [2,14]. This indicates that SSc has a shared immune pathogenesis with other autoimmune diseases providing further support for the concept of quantitative thresholds in immune-cell signaling. In this concept, several genetic factors of relatively small effect may cumulatively create a state of susceptibility to autoimmune diseases (reviewed in [15]). Self-reactive B and T-cells are a normal component of the immune system. However, they are usually kept in check by regulatory mechanisms in the thymus/bone marrow or peripheral blood. In the concept of quantitative threshold, the implicated genetic variations lead cumulatively to an impairment of necessary biological processes for destruction of self-reactive immune cells and regulating auto-reactivity. Validity of this concept in SSc is supported by the fact that several SSc genetic susceptibility loci overlap not only with SLE but also with other autoimmune diseases. For example, *STAT4 *is also implicated in rheumatoid arthritis [16], and primary biliary cirrhosis [17]. Similarly, *PTPN22 *is a susceptibility locus in rheumatoid arthritis [18], type 1 diabetes mellitus [19], and also SSc [20].

**Table 1:** Selected non-major histocompatibility complex susceptibility genes for systemic sclerosis which were confirmed in at least two independent studies.

	Approximate OR	Potential Function
BANK1	1.2-1.5^*^	Lymphocyte activation - B cells
BLK	1.2-1.5	Lymphocyte activation - B cells
CD247	1.2-1.5^*^	Lymphocyte activation - T cells
CD226	1.0-1.2	Lymphocyte activation - T cells
IRF5	1.2-1.5	Innate immune signaling - Interferon pathway
MIF	1.0-1.2	Adhesion molecule on endothelial cells
PTPN22	1.2-1.5	Lymphocyte activation - T cells
STAT4	1.2-1.5	Innate immune signaling and lymphocyte activation - T cells
TNFSF4	1.2-1.5	Lymphocyte activation - T cells and B cells

^*^The minor allele is protective. The inverse ratio OR is reported.

Some of the confirmed SSc susceptibility loci show a stronger association with its serological or clinical (limited versus diffuse) [13] subtypes than the overall disease. Several genetic associations in the HLA [8,21] or non-HLA regions, such as *BANK1, IRF8, SOX5 and IRF7 *are mainly with the SSc-related autoantibodies (e.g. anti-centromere or anti-topoisomerase I) or clinical subtypes of disease [1,2,8,22]. Furthermore, many of the identified single nucleotide polymorphisms (SNPs) are merely a tag genetic variant for the yet to be identified causal allele. This is also applicable to GWA studies, because the utilized platforms provide more than 80% coverage for common polymorphisms in human genome by investigating SNPs that are in strong linkage disequilibrium with multiple other SNPs and serve as proxies for gene areas. Advances in gene sequencing techniques will permit large scale sequencing of these susceptibility genes to pinpoint the actual causal variant.

Some of the reported genetic associations in one ethnic group might not replicate in other ethnicities. The reported polymorphisms might not tag the causal locus in all ethnic groups because of the varying linkage disequilibrium structure among different ethnicities. Alternatively, the reported genetic associations might be truly an ethnic specific susceptibility locus for SSc.

It is noteworthy that the gene variants of interest do not operate in isolation as they are parts of intertwined biological pathways. Therefore, examination of gene-gene or gene-environment interactions can lead to better understanding of SSc pathogenesis. Lastly, mechanistic studies are needed to elucidate how these immune system gene variants contribute to the cross-talk among immune, vascular and fibrotic pathways leading to the unique phenotype of SSc.

### Implication of SSc genetics for predicting disease severity and organ involvement

SSc is associated with high morbidity and mortality. The disease related mortality is mainly driven by internal organ involvement [23], especially severity of lung disease [24,25]. As shown in Table 2, several studies have also investigated the association of MHC and non-MHC genetic loci with interstitial lung disease (ILD), pulmonary arterial hypertension (PAH), scleroderma renal crisis, and mortality. It is important to point out that the comparison of SSc patients with a particular disease manifestation with patients without that particular organ involvement (case-case analysis) is more relevant for biomarker development than comparison of patient with the disease manifestation to unaffected controls (case-control analysis). The main reason for this notion is that the prognostic biomarkers are useful if they can aid clinicians to subgroup patients (cases-case analysis) based on the expected disease progression. A case to control comparison does not occur in the clinical settings because the diagnosis of SSc is already established before clinicians become interested in predicting the disease course. *IRF5 *gene variants have been linked to overall mortality independent of disease type and serology [26]. *CTGF *[27], *HGF *[28], *IRAK1 *[29], *IRF5 *[6,26,30], *MMP-12 *[31], *SP-B *[32] polymorphisms are reported to be associated with ILD. The case definition for ILD varies considerably, some investigators have relied on the presence of reticular or ground glass opacities on high resolution chest computer tomography (HRCT) while others have focused on severity of ILD based on the pulmonary function results. The former approach does not differentiate between the mild stable ILD and its severe progressive forms. Furthermore, *IL23R *[33], *KCNA5 *[34], *TLR2 *[35], *TNAIP3 *[36], and *UPAR *[37] genes are reported to be associated with PAH while HLA-DRB1*04:07 and *13:04 were associated with scleroderma renal crisis [38].

**Table 2:** Selected genes associated with various SSc disease manifestations based on case- case comparisons.

Gene	Clinical Association
CTGF	ILD by pulmonary function test or HRCT [27]
HGF	End-stage ILD by pulmonary function test [28]
HLA-DRB1*04:07	Scleroderma Renal Crisis [38]
HLA-DRB1*13:04	Scleroderma Renal Crisis [38]
IL23R	PAH by echocardiogram or right heart catheterization [33]
IRAK1	ILD by HRCT [29]
IRF5	Overall survival [26] ILD by HRCT [6,30] Severity of ILD by pulmonary function test [26]
KCNA5	PAH by right heart catheterization [34]
MMP-12	ILD by pulmonary function test and HRCT [31]
SP-B	ILD by HRCT [32]
TLR2	PAH by right heart catheterization [35]
TNFAIP3	PAH by right heart catheterization [36]
UPAR	PAH by right heart catheterization [37]

However, the above findings need to be replicated in independent studies. Furthermore, the currently available cross-sectional patient populations for SSc genetic studies are most likely affected by survival bias, i.e. the examined prevalent cohorts with longstanding disease are depleted of patients with the most progressive and severe form of SSc. For example, SSc patients with rapidly progressive ILD have a higher mortality [39], therefore patient samples with long-standing disease (mean disease duration > 5 years) are depleted of the most severe form of ILD. This can lead to decreased frequency of genetic loci associated with more severe forms of disease in the investigated patient samples. Examination of incident cases with longitudinal follow-up can avoid problems arising from survival bias. Furthermore, the genetic severity loci might be different than the genes linked to SSc susceptibility. For example, HGF was not a susceptibility locus for SSc but was associated with end-stage lung disease among Japanese SSc patients [28]. A careful phenotypic characterization of patients examined in GWAS can permit an unbiased profiling of severity loci. This will also allow combination of genetic data with other clinical and serological markers of disease severity for risk prediction.

Risk prediction in genetically complex diseases like SSc requires statistical approaches that extend beyond separate odds ratios for each SNP of interest. Genotypes at multiple SNPs can be combined into cumulative scores calculated according to the number of severity alleles carried. Furthermore, risk reclassification statistics can be used to combine genetic and clinical data. In this approach, patients in the intermediate risk group based on clinical data are reassigned to low- or high-risk categories using the pertinent genetic information.

### Implication of SSc genetics for treatment selection

The newly identified genetic susceptibility pathways can lead to identification of novel therapeutic targets and guide drug development. Indeed, some of the currently investigated biologic therapies for SSc match appropriately to these pathways. These include anti-interferon (e.g. sifalimumab) and anti-B-cell agents (e.g. rituximab) [40]. Furthermore, the SSc genetic data lend support to T-cell directed therapies (e.g. abatacept). However, there are no reported large-scale, randomized controlled studies of B-cell, T-cell, interferon directed therapies in patients with SSc.

Beyond identification of new therapeutic targets, the genetic information might be used to identify the high responsive group to a particular biologic treatment. There are no data on predictive significance of genetic information for response to treatment in SSc. This requires the collection of genetic material in drug trials and careful analysis of genetic information conditional on the study outcomes. Considering the modest effect of these gene variants on the disease susceptibility, we might be underpowered to examine the predictive significance of these factors in drug trials using traditional (frequentist) statistical methods (especially after sample partitioning into treatment and control arms). Bayesian analysis of trial results in uncommon diseases such as SSc [41] might lead to more flexible and clinically useful biomarker development.

Independent of disease susceptibility genes, the genetic information can be used to predict drug metabolism and development of adverse effects (pharmacogenetics). For example, polymorphism in the *UGT1A9 *affect metabolism of mycophenolate mofetil and predict acute rejection in renal transplant patients [42,43]. Despite the widespread use of mycophenolate mofetil, the role of this polymorphism for response to treatment and development of adverse events has not been investigated in SSc patients.

In a recently published study, a polymorphism in the *IL-6 *gene predicted response to rituximab in a sample of patients with SLE and other rheumatic diseases that included patients with SSc [44].

## Conclusion

The significant advances in SSc genetics represent an opportunity for biomarker development. Careful phenotypic characterization, independent confirmation of current findings, inclusion of genetic studies in drug trials, and utilization of novel analytic approaches paired with advanced high-throughput technologies can potentially lead to identification of genetic markers that predict disease severity and response to treatment in SSc.

## Abbreviations

GWAS: Genome wide association studies; HLA: Human leukocyte antigen; HRCT: High resolution chest computer tomography; ILD: Interstitial Lung Disease; MHC: Major histocompatibility complex; PAH: Pulmonary arterial hypertension; SNP: Single nucleotide polymorphism; SSc: Systemic sclerosis.

## Competing interests

The authors declare that they have no competing interests.

## Authors' contributions

SA, TR, MM, and JM were involved in drafting and revising of the manuscript and approved its final version. All authors meet the criteria for authorship.

## Authors' information

SA is associate professor of medicine/rheumatology at the University of Texas-Houston (USA). His research focuses on correlation of genomic data with important clinical outcomes in systemic sclerosis and other rheumatic diseases.

TR is a professor of Rheumatology & Clinical Immunology at the University of Utrecht (The Netherlands). His area of research focuses on mechanistic and genetic translational studies in systemic sclerosis and other rheumatic diseases.

MM is a professor of medicine/rheumatology at the University of Texas-Houston (USA). Her research focuses on genetic and clinical studies in systemic sclerosis.

JM is a professor of genetics in Instituto de Parasitología y Biomedicina López-Neyra, Consejo Superior de Investigaciones Científicas (CSIC) in Granada (Spain). His research focuses on genetics of systemic sclerosis, as well as other rheumatic and autoimmune diseases.

## Pre-publication history

The pre-publication history for this paper can be accessed here:

http://www.biomedcentral.com/1741-7015/11/9/prepub
